# Speech therapy students’ perceptions of authentic video cases in a theory module on child language disorders

**DOI:** 10.4102/sajcd.v66i1.602

**Published:** 2019-01-22

**Authors:** Helena Oosthuizen

**Affiliations:** 1 Division of Speech-Language and Hearing Therapy, Department of Rehabilitation Sciences, Faculty of Medicine and Health Sciences, Stellenbosch University, South Africa

## Abstract

**Background:**

Undergraduate speech-language therapy students often find it difficult to see the relevance of theoretical module content, which may negatively influence their motivation to learn. The real world of their future profession can be brought to life in the theory classroom by including authentic case study examples. Video case studies are well suited to illustrating communication disorders and may also be easier to remember and relate to information already in the long-term memory.

**Objectives:**

This article describes the perceptions of undergraduate students regarding the inclusion of authentic video cases in a theoretical module on developmental communication disorders.

**Methods:**

A qualitative, interpretivist research design was followed. Focus-group interviews were conducted with 22 second-year students in the programme B Speech-Language and Hearing Therapy. A modified contextualised content analysis approach was used to analyse interview data.

**Results:**

The use of authentic video cases was perceived positively by participants. Seeing a realistic example of a person with communication difficulties made it easier to understand, remember and engage with the module content. Participants also felt they could more easily imagine themselves in that clinical context, which seemed to (re-) awaken in them a sense of purpose and motivation. Being presented with real-life communication problems made them realise the relevance of their profession. However, participants experienced cognitive overload at times when the processing requirements of a task exceeded their available cognitive capacity.

**Conclusion:**

Video cases are valuable tools to enhance students’ engagement with theoretical content. To avoid cognitive overload, a scaffolded multimedia learning experience needs to be provided.

## Introduction

**FIGURE 1:**
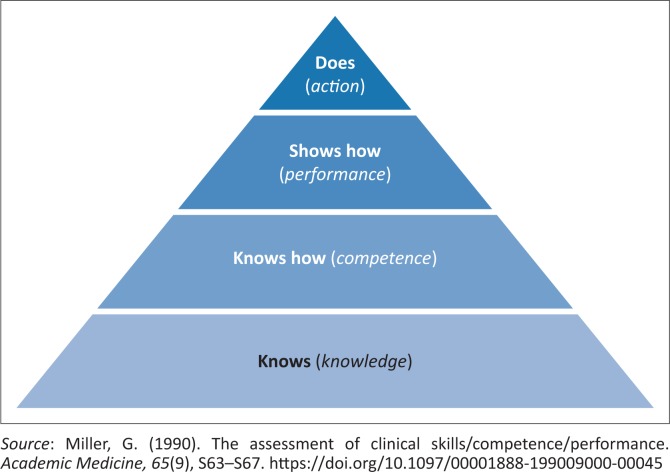
Miller’s prism of clinical competence.

Undergraduate students in the field of speech-language therapy often find it difficult to apply their theoretical ‘book knowledge’, in other words, to demonstrate competence in a clinical context (Hoben, Varley, & Cox, 2007). During their early study years, the main focus is on obtaining the theoretical basis needed for clinical reasoning and problem-solving – that is, the ‘know’ and ‘know how’ levels of Miller’s prism of clinical competence (Miller, 1990) presented in Figure 1 – and students typically venture onto clinical training platforms only after completing the relevant theory. The relevance of theoretical modules may therefore not always be clear to pre-clinical students. This can negatively influence their motivation to learn (Kember, Amber, & Hong, 2008). The real world of their future profession can, however, already be brought to life in the theory classroom by including authentic case study examples (Zipp & Maher, 2010).

Research on multimedia learning suggests that, under certain circumstances, ‘people can learn more deeply from words and pictures than from words alone’ (Mayer, 2014). Presenting information as words (e.g. textbooks) along with pictures (e.g. videos) may facilitate deeper learning as students attempt to create associations between the two types of input. Visual representations may also be easier to remember and relate to information already in the long-term memory (Cherney, 2008; De Leng, Dolmans, Van de Wiel, Muijtjens, & Van der Vleuten, 2007). If, however, one assumes that humans process verbal and visual material through different channels (dual-channel assumption), each of which has limited processing capacity (limited capacity assumption), the cognitive load of a multimedia learning opportunity needs to be carefully managed. Otherwise, the processing requirements of a task may exceed a student’s available cognitive capacity and lead to cognitive overload (Mayer & Moreno, 2003).

Video case studies are particularly well suited to illustrating communication disorders, because they allow students to temporarily distance themselves from the real-time requirements of communication interaction and free up cognitive resources needed to engage with language as an object of study. Also, because language is an abstract mental capacity, having a permanent record of a communication interaction with a language-impaired person is valuable to the novice not yet skilled in observing short-lived language behaviour. Video clips from websites such as YouTube are commonly used in lectures but these are seldom recorded with specific educational outcomes in mind and usually depict situations and behaviour that have little in common with the South African context. These limitations are especially problematic when considering the critical role that context and culture play in communication. Against this background, and given that no studies could be sourced on how pre-clinical speech therapy students experience real video cases, funding was obtained to develop a database of authentic case studies of South African children with communication impairment and to pilot its implementation with students. The aim of this article is to describe how students perceived the video cases after they were exposed to them in a theory module on developmental communication impairment.

## Literature

Benefits of using video cases in health sciences education range from enhancing memory for course content (Cherney et al., 2008; De Leng et al., 2007), improving clinical reasoning skills (Hoben et al., 2007; Lysaght & Bent, 2005), stimulating shared cognition in a problem-based learning curriculum (Balslev, De Grave, Muijtjens, Eika, & Scherpbier, 2005), and increasing student motivation (Kember et al., 2008). Authentic video cases have also been incorporated in speech therapy training programmes in the UK and USA. For instance, the UK-developed online repository of case studies known as Patient Assessment Training System (PATSy) contained ‘virtual’ patient cases in several clinical domains including speech and language (Cox, & Lum, 2004). This system made it possible for students to access raw assessment data and background information about real clients and to systematically test hypotheses about clients’ underlying cognitive processing difficulties, thereby improving their clinical reasoning skills. Similarly, Baharav (2008) reported on the development of a searchable database of video-recorded clinical sessions at the training clinic of Western Washington University and its application in undergraduate student training. Although the aim of these two papers was not to describe undergraduate students’ perceptions of being exposed to real cases, both reported positive student feedback.

Medical students’ views on the added value of video cases in comparison to paper cases in a problem-based learning curriculum were examined in a qualitative study by De Leng et al. (2007). Findings from focus-group interviews indicated that video cases had several perceived benefits, such as: (1) being more true to life than text cases and therefore easier to visualise, (2) being memorable, (3) being motivating and making it easier for students to imagine themselves in certain situations and (4) representing a rich source of information. Factors that hindered learning for these participants were videos that were too long and in which not enough structure was provided by the tutor. This study illustrates the potential value that video cases can add to students’ training and also highlights the importance of taking into account students’ perceptions when devising learning opportunities. Based on the available literature, it was expected that inclusion of video cases would encourage deeper learning in the sense that participants in this study would have a better understanding of the module content, increased awareness of specific clinical populations and enhanced motivation for their studies.

## Methods

The study followed a qualitative, interpretivist research design, considered appropriate for the purposes of this study because it entails using participants’ ‘words and descriptions to record and investigate aspects of social reality’ (Bless, Higson-Smith, & Sithole, 2013). Focus-group interviews were conducted with second-year students in the programme B Speech-Language and Hearing Therapy at Stellenbosch University. Focus-group interviews are considered an effective tool to explore subjective, emotional experiences (Tracy, 2013) and were therefore considered appropriate for this study.

### Context

Participants were enrolled in a 7-week module on communication disorders in specific clinical populations. A total of seven different topics were addressed during 3-h-long weekly lectures (see Table 1). During the first hour, students wrote a short multiple choice questionnaire (MCQ) test on the preparatory reading, watched one or two video cases ranging between 8 and 20 min and/or studied the written case history information. Thereafter, students received a guest lecture by a speech therapist with clinical expertise in the particular area. During this 2-h lecture, theoretical information was discussed and related to the specific case study viewed in the video. Cases had been developed in collaboration with the guest lecturer in order to provide students with ‘typical’ examples of the specific clinical population. The video cases depicted the child in an everyday context during an intervention session (assessment and/or therapy) and were edited by the author to depict the most salient aspects of the session in a shortened format. Subtitles in English were provided for all child utterances, as well as for any Afrikaans utterances used by the therapist.

**TABLE 1:** Lecture topics and description of presented cases.

Topic number	Topic	Cases†	Length of video
1	Intellectual disability	Leah and Ismail	22 min
2	Children in institutionalised care‡	Anathi	-
3	Paediatric HIV and/or AIDS	Funeka	12 min
4	Auditory processing disorder‡	Nicky	-
5	Autism spectrum disorders	Emily and Kiaan	34 min and 10 min
6	Visual disabilities	Babalwa and Eva	18 min and 16 min
7	Language learning disorder	Irvin and Kyle	13 min and 17 min

† , Pseudonyms;

‡ , Video cases could not be obtained for topics 2 and 4 because of ethical and practical challenges.

### Participants

Participants were selected using purposive sampling. All 30 students who were enrolled in the module in which the videos were piloted were invited to participate in the study at the end of the module. Students were recruited via email and followed up with a text message inviting them to take part in the study and 22 students agreed to take part (male = 1; female = 21; mean age 20 years). Two focus groups were conducted with 9 and 10 students, respectively, in the language preferred by the group (Afrikaans and/or English). Three participants who were not able to attend the scheduled focus group interviews because of unforeseen events were later interviewed individually. In all interviews, an adaptive interview schedule consisting of standardised open-ended questions was used to guide the discussion and ensured consistency between interviewers. Questions were related to themes identified in the literature review on students’ experiences of video cases. Interviews were conducted by two independent persons not involved in students’ clinical training, namely a speech therapist and a PhD student in clinical linguistics, both of whom have experience in qualitative interviewing and are fluent in both Afrikaans and English. Although the same interviewers conducted the focus group and individual interviews using a standardised interview schedule, it is acknowledged that differences in interpersonal dynamics in the two data collection methods may have influenced the nature of responses obtained.

### Data analysis

A modified contextualised content analysis approach was used to identify themes addressed by participants. To ensure reliability, the following audit trail was established: all interviews were orthographically transcribed by the author and the first interviewer. Verbal and written feedback was obtained from both interviewers after completion of the interviews, highlighting key aspects of the interview process. The author further immersed herself in the data by reading and re-reading the transcripts and listening to the interviews, and made research memos to record personal insights related to certain sections of text. During this process, several recurring themes were identified and further grouped into four main themes that were coded into the data using Atlas.ti software. To enhance the authenticity of findings, a member check was conducted. All participants were provided with the written transcript of the interview, as well as the preliminary results, and invited to approach the researcher if there were any disagreements regarding interpretation of the data.

### Ethical consideration

Ethical approval was obtained from the health research ethics committee of the Faculty of Medicine and Health Sciences (N15/10/113) and the Unit for Institutional Research and Planning at Stellenbosch University. Permission to collect case study information was obtained from the Western Cape Departments of Education and Health (20161027–5599). Informed consent was obtained from all participants. All identifying personal information was removed from the written and video cases during editing, and from the interview transcripts, to protect the participants’ privacy. No personal details of participants were revealed to anyone not directly involved in the study. Data were saved in a password-protected computer file to which only the researchers had access. Videos were shown only to the students enrolled in the module and were not available for them to download or distribute.

## Results

**FIGURE 2:**
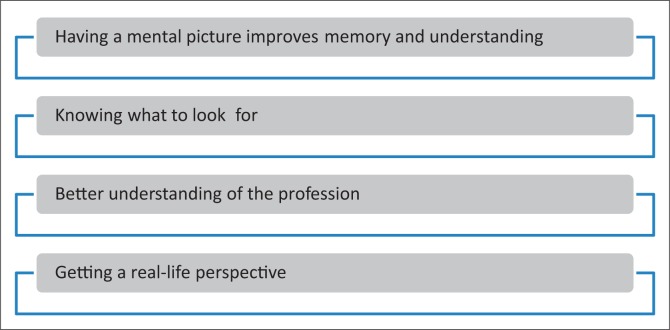
Themes identified from focus group discussions on the inclusion of video cases in a theory module in an undergraduate speech therapy curriculum.

Findings from the two focus groups and three individual interviews will be discussed together according to the main themes outlined in Figure 2. Selected quotations are used throughout to illustrate themes in participants’ own words.

### Having a mental picture improves memory and understanding

‘But for the things that I saw, I can speak about that.’ (Focus group 1, female)

‘And now I understand what they say in the lecture, because I could see it.’ (Focus group 1, female)

The majority of students were of the opinion that video cases made the theoretical module content more memorable, because they now had a mental image that they could refer back to. Having a real example helped them to more fully understand and engage with the theory, and several students recommended that real video cases already be used in first year. Students also reported increased awareness of the communication characteristics of the clinical populations studied and knew better what to expect from their clinical training site the following year. However, they did not yet feel confident in their ability to actually do intervention.

### Knowing what to look for

‘Or even if they were to show us the whole long video, then just like stop it every five minutes and say, “Did you see this and this and this? And now look out for this and this and this”. So just to guide your thoughts, so that you’re forced to focus as well.’ (Focus group 1, female)

‘She’d like say okay um they’re prone to echolalia and then she shows us, this is what echolalia is, then she shows three or four, five second videos, then you’re like okay that makes sense now. Instead of saying, remember in the video at the beginning or halfway through then you’re like…no. So yes, it just helps to have a referencing point.’ (Focus group 1, female)

Students felt they would have been able to learn more from the video cases if they had had a better idea of what they were expected to focus their attention on, especially with longer videos. They felt they learned much better and were more engaged with the content when short video clips were integrated into the lecture to demonstrate important concepts, as this helped them to form associations between the theory they heard or read and what they saw. Another way in which students felt the lecturer could better scaffold their learning experience was to let them view the video as a whole but to first give a short explanation and point out specific aspects to look out for. They also expressed a need to see more varied examples of each clinical population for a broader overview and in different contexts.

### Better understanding of the profession

‘And you see the challenges that they [clients] face and then you realise that as a future therapist you have to be able to do and the skills that you need to have and how you need to go about to facilitate the development of those individual skills.’ (Focus group 2, female)

‘When I watched the videos I asked myself about that one blind boy […] are you emotionally ready for your profession to basically work with these populations? So I think it just sparks that idea of, listen here, you’re on your way somewhere to actually go and make a difference.’ (Focus group 2, male)

Participants found it very motivating to be exposed to real video cases, because they felt it gave them a better understanding of what their future profession entails and the type of clinical problems they will encounter. Given the fact that second-year students still have relatively limited clinical exposure, they valued seeing the ‘bigger picture’ of what they were working towards in their studies. Importantly, having an authentic record of a client–therapist encounter caused many students to identify and reflect on key interpersonal skills (such as patience) needed to be an effective therapist. This allowed them to construct a more realistic view of their chosen profession, their personal preferences (e.g. wanting to work with children) and the personal growth required of them.

### Getting a real-life perspective

‘It’s not just a piece of paper. You get to visually watch the videos and see the actual children and see the actual lecturers giving therapies. It makes a difference to see the techniques and the children.’ (Focus group 2, female)

‘I guess you kind of take it more seriously because it’s like a real thing. This isn’t like a fictional case or whatever, you know, this is like a real person who has gone through these real things, you know, like it’s kind of like you take it more seriously when dealing with people’s lives.’ (Focus group 1, female)

Seeing video cases of actual therapists interacting with actual children with communication impairment made students realise the real-life implications of the content they were dealing with. Although the main goal of viewing the videos were not to analyse the skills of the therapist, students nevertheless viewed the therapist as a role model in interacting with the child. The students also enjoyed the fact that, in most cases, the therapist in the video was also the guest speaker who presented the lecture and that they could learn from them in person.

## Discussion

The use of authentic video cases in an undergraduate theory module was generally perceived very positively by participants in this study. Seeing a realistic example of a person with communication difficulties, reportedly made it easier to understand, remember and engage with the module content. This benefit of having a mental picture to refer back to echoes findings of previous studies (e.g. Cherney, 2008; De Leng et al., 2007) and is in line with the multimedia principle, namely that deeper learning is possible when learning through words and pictures than when learning from words alone (Mayer, 2014). However, it is clear from the findings that unguided interaction with the videos limited the value of the experience for students. When faced with the rich source of information that a video case represents, pre-clinical students can easily feel lost or overwhelmed. As second-year students do not yet have an established knowledge base in long-term memory to guide their viewing of the videos, they need to mentally hold and manipulate the images and words in their working memory, which has limited capacity (Mayer & Moreno, 2003). Students’ feedback seems to suggest that they experienced cognitive overload at times, either because of the length of the video, the perceived lack of guidance, the timing of the module or a combination of these factors. To avoid this, lecturers should consider incorporating short video clips into a traditional lecture format or providing clear instructions before students view the video case.

The purpose for which a video case is used is likely to influence the learning outcome achieved. Participants in this study felt that video cases fostered a greater awareness of the clinical characteristics of different clinical populations, but did not equip them with the practical skills they needed for intervention. To an extent, this finding was to be expected, because the focus of the module was largely introductory in nature and is followed up in third year with a year-long, dedicated clinical training site during which specific clinical skills are acquired. The exposure to authentic video cases seemed nevertheless to be highly motivating to students, partly because it gave them a better understanding of the extent and relevance of speech therapy. In line with findings by De Leng et al. (2007), participants also felt they could more easily imagine themselves in that clinical context, which seemed to (re-)awaken in them a sense of purpose and motivation. Seeing the range and extent of communication difficulties they will one day encounter, led them to critically reflect on the skills they would need to be an effective therapist. In that sense, the video case presented them with a valuable role model in the form of the therapist interacting with a client in a real-life clinical encounter (De Leng et al., 2007).

## Conclusion

The inclusion of authentic video cases in an undergraduate theory module was generally perceived positively by the participants in this study, who felt that theoretical module content was easier to understand and remember when one had a mental image to refer back to. Being presented with real-life communication problems made them realise the relevance of their profession and reflect on the skills they would need to cultivate to be effective therapists. In order to avoid cognitive overload, a scaffolded multimedia learning experience needs to be provided which takes into account students’ working memory and processing limitations. This study presents the subjective views of a specific group of participants at a particular point in time and needs to be followed up with more objective measures to determine if and to what extent the inclusion of video cases influences student performance.
